# 

**Published:** 1901-11

**Authors:** 


### MARRIAGES.

At Naco, Arizona, October 1, 1901, Dr. Wm. G. Shaw, formerly of Philadelphia, was married to Miss Claudia Murray, formerly of New York City. Dr. Shaw is an attaché of the Bureau of Animal Industry, a graduate of the Veterinary Department of the University of Pennsylvania, and late house surgeon of the Veterinary Hospital of the University of Pennsylvania.

Dr. Edgar W. Tully, of Philadelphia, was married November 19th to Miss Louise Fawcett Peltz, of Philadelphia, at the Baptist

Temple. Dr. Tully is a graduate of the Veterinary Department of the University of Pennsylvania, class of 1890, and has also a degree in human medicine, which he has practised for several years.

### PERSONALS.

Dr. E. H. Lenhert, of Clinton, Mass., has succeeded Dr. Nelson S. Mayo at the Connecticut Agricultural College, Storrs, Conn., as professor of anatomy and veterinary science.

Dr. W. E. Wight, of Pittsburg, is reported as an applicant for the position of city veterinarian under the new administration now in power in that city.

Dr. H. L. B. Coote, formerly of Chicago, is now a resident of Michigan City, Ind.

Dr. Dennish, of Morrisville, Pa., is entering the livery-stable business in Trenton, N. J.

Dr. M. H. McKillip has been appointed chief veterinary inspector for the Chicago horseshow to be held in November. To act as his assistants, Dr. Gerald E. Griffin, United States Army, and Dr. Orion E. Dyson, of Chicago, have been selected. The Association has already been assured of the most successful show ever held in America.

Dr. J. C. McNeil, of Pittsburg, Pa., has been retained as city veterinarian by the new administration in power in that city.

Dr. R. A. Phillips, veterinary surgeon of Jackson, Miss., is a matriculate of the Kansas City Veterinary College.

Dr. J. A. Courtright, of Clark's Green, Pa., has been engaged to take a transport to South Africa for the British Government from New Orleans, La.

Dr. A. H. Fehr, of Canandaigua, N. Y., formerly a student of the Ontario Veterinary College, is a matriculate at the McKillip Veterinary College, session of 1901–1902.

Dr. R. W. English, successor to the late Dr. W. H. Arrowsmith, of Jersey City, has a commodious hospital in connection with his practice. Dr. English has a place for a graduate as an assistant and house surgeon.

Dr. N. B. Rhodes, of Tampa, Fla., has a complete sanitarium for his patients, which he conducts in connection with his practice.

Pittsburg veterinarians are watching with much interest the suit of Dr. D. C. Gearhart for a large claim for professional services and expenses incurred in searching for and the selection of a fine coach team for one of the " Smoky City's " leading politicians.

Dr. Thomas Trinder, of Charlotte, N. C., is a matriculate of the McKillip Veterinary College.

Dr. W. Ratcliffe, of Waynesburg, Green county, Pa., has announced his retirement from active practice of veterinary medicine in his county.

Dr. John R. Mohler, of the Bureau of Animal Industry, located at Washington, D. C., returned to Philadelphia during election week.

Dr. E. H. Flood, of Philadelphia, Pa., is one of the best-posted veterinarians in life insurance in the profession.

Dr. E. C. Lahr, of Kansas City, Mo., has removed to Sabetha, Kansas, where he will continue to practice in veterinary medicine.

Dr. James Fitzgerald, of Monroe, N. Y., while performing professional duties in the mountainous district of New York, was seriously injured by the harsh treatment of some ruffians.

Dr. T. D. Skeen, formerly of Garland, Ill., is now practising at Blackwell, Oklahoma Territory.

Dr. C. J. Marshall, of Philadelphia, fills the rôle of veterinarian to the Pediatric Society, an organization doing much to improve the milk-supply of Philadelphia.

Dr. E. H. Nodyne, of Sodus, N. Y., fills the rôle of Manager of the Sodus Coach-horse Company.

Drs. J. W. Sallade, of Pottsville, and W. Horace Hoskins, of Philadelphia, were delegates to the Democratic State Convention in August.

Veterinarian Major Massie, of the Royal Canadian Field Artillery, of Kingston, Canada, has just returned from a trip to South Africa with a brigade division of Canadian artillery.

Dr. Charles Bland, of Falls of Schuylkill, Philadelphia, Pa., met with the great misfortune of the loss of a son, thirteen years old, by drowning, in July.

Dr. L. Van Es, of Mobile, Ala., stopped in Philadelphia for some hours on his way to Atlantic City.

Dr. W. B. E. Miller, of Camden, N. J., was seriously hurt in a trolley accident in August.

Dr. L. A. Nutting, of Syracuse, N. Y., has entered the Bureau of Animal Industry as assistant meat-inspector.

Dr. N. P. Whitmore, of Gardner, Ill., met with the loss of one of his children after twenty-four hours' illness from cerebro-spinal meningitis.

Dr. Leonard Pearson, State Veterinarian of Pennsylvania, was at the Hanover Veterinary College in the latter part of July.

The Governor of New Jersey has appointed two veterinarians on the Good Roads Commission—Drs. William Herbert Lowe, of Paterson, and T. Earle Budd, of Orange.

Dr. J. B. Cosgrove, of Worcester, Mass., who has been ill for a long time, is again at his post of active professional duties.

Dr. S. B. Willard, of Yardley, Pa., has just been appointed postmaster for that town, which is one of the oldest settlements in Eastern Pennsylvania.

